# Control Work

**Published:** 1901-09

**Authors:** 


### CONTROL WORK.

At the meeting of the Minnesota State Board of Health, July 10, 1901, Dr. S. D. Brimhall was chosen director of the Veterinary Department. Since the transfer of this department to the office of the State Board of Health, August, 1900, Dr. Brimhall has been performing the duties of director, although not bearing this title.

Various rules were presented to the board looking to the prevention of the importation of animals suffering from contagious diseases, such as tuberculosis, glanders, sheep scab, etc.

A very interesting report from Dr. S. D. Brimhall, of the Veterinary Department, and Dr. L. B. Wilson, of the Bacteriological Laboratory of the board, was presented upon sixty-four cases of hemorrhagic septicæmia in cattle.

