# Correction

**Published:** 1901-10

**Authors:** 


### CORRECTION.

DEATH OF DR. SHAUB. In the September issue of the JOURNAL, in referring to the death of Dr. A. E. Shaub, of Lancaster, Pa., we inadvertently associated the ownership of the veterinary hospital and practice of Dr. J. C. Shaub, which led some of the latter's friends and acquaintances to think he was the one who had died.

